# Influence of Morphine on Pharmacokinetics and Pharmacodynamics of Ticagrelor in Patients with Acute Myocardial Infarction (IMPRESSION): study protocol for a randomized controlled trial

**DOI:** 10.1186/s13063-015-0724-z

**Published:** 2015-04-29

**Authors:** Jacek Kubica, Piotr Adamski, Małgorzata Ostrowska, Marek Koziński, Karolina Obońska, Ewa Laskowska, Ewa Obońska, Grzegorz Grześk, Piotr Winiarski, Przemysław Paciorek

**Affiliations:** Department of Cardiology and Internal Medicine, Nicolaus Copernicus University, Collegium Medicum, 9 Skłodowskiej-Curie Street, 85-094 Bydgoszcz, Poland; Department of Principles of Clinical Medicine, Nicolaus Copernicus University, Collegium Medicum, 9 Skłodowskiej-Curie Street, 85-094 Bydgoszcz, Poland; Department of Pharmacology and Therapy, Nicolaus Copernicus University, Collegium Medicum, 9 Skłodowskiej-Curie Street, 85-094 Bydgoszcz, Poland; Dr. J. Biziel Memorial University Hospital No. 2, 75 Ujejskiego Street, 85-168 Bydgoszcz, Poland

**Keywords:** Ticagrelor, Morphine, Myocardial infarction, Pharmacokinetics, Pharmacodynamics

## Abstract

**Background:**

Ticagrelor is an oral platelet P2Y12 receptor antagonist which is recommended for patients suffering from myocardial infarction, both with and without persistent ST segment elevation. Morphine is the first choice drug in pain alleviation in the same clinical subset. Recently a possible negative influence of morphine on the pharmacokinetics and antiplatelet effects of P2Y12 receptor blockers has been postulated.

**Methods/design:**

The IMPRESSION study is a phase IV, single center, randomized, double-blind, placebo-controlled clinical trial that is designed to assess the influence of morphine on the pharmacokinetics and pharmacodynamics of ticagrelor in patients with myocardial infarction. The study is planned to include up to 100 patients with myocardial infarction who will be randomized into one of two arms in a 1:1 ratio. Subjects in the intervention arm prior to the loading dose of ticagrelor (180 mg) will receive morphine (5 mg) intravenously, whereas patients in the control arm will receive a placebo prior to the loading dose of ticagrelor (180 mg). The pharmacokinetics of ticagrelor and its active metabolite (AR-C124910XX) will be assessed by liquid chromatography mass spectrometry. Platelet function testing in each patient will be performed using up to four different methods (platelet vasodilator-stimulated phosphoprotein assay, multiple electrode aggregometry, VerifyNow, and light transmission aggregometry).

**Discussion:**

This study is expected to provide essential evidence-based data on the impact of morphine on the absorption of ticagrelor in patients with myocardial infarction as well as to shed some light on the suspected connection between morphine use and antiplatelet activity of ticagrelor in the same group of patients.

**Trial registration:**

ClinicalTrials.gov identifier: NCT02217878 (14 August 2014).

## Background

Platelet activation and aggregation play a pivotal role in the pathophysiology of acute coronary syndromes (ACS), including ST-segment elevation myocardial infarction (STEMI) and non-ST-segment elevation myocardial infarction (NSTEMI). Dual antiplatelet therapy, consisting of aspirin and one of the P2Y12 receptor antagonists, constitutes the mainstay of ACS treatment. Achievement of adequate and timely platelet inhibition is of vast importance in the acute phase of myocardial infarction. Results of the PLATO trial have shown that therapy with ticagrelor, a novel platelet P2Y12 receptor inhibitor, compared with clopidogrel reduces not only the rate of death from vascular causes, myocardial infarction, or stroke, but also all-cause mortality, without concomitant increase in the rate of overall major bleeding, in patients with ACS [1,2]. The superiority of ticagrelor over clopidogrel evidenced in the PLATO trial has been principally attributed to ticagrelor’s fast, potent, and uniform pharmacodynamic features [2,3].

Based on the mentioned results, therapy with ticagrelor received class I B recommendation according to the European Society of Cardiology (ESC) in interventionally treated STEMI patients and in all moderate-to-high risk patients with ACS without persistent ST-segment elevation, including NSTEMI patients, regardless of initial treatment strategy [4,5]. Likewise, the American College of Cardiology Foundation and American Heart Association (ACCF/AHA) Task Force on Practice Guidelines recommend the use of ticagrelor in these subsets of patients [6,7].

The same ESC and ACCF/AHA guidelines recommend the use of morphine as a treatment of choice for pain relief in the clinical setting of myocardial infarction [4-6]. However, these recommendations, although strong, are only based on expert consensus. Pain relief in patients presenting with acute myocardial infarction (AMI) is suggested to lead to decreased sympathetic activation, thus preventing vasoconstriction and subsequent increase in the workload of the heart. Additionally, apart from its analgesic effects, morphine also alleviates the work of breathing and reduces anxiety [4,6]. On the other hand, despite its favorable analgesic and sedative actions, morphine also exerts adverse effects, which include hypotension, bradycardia, respiratory depression, vomiting, and reduction of gastrointestinal motility. Importantly, both the safety and efficacy of morphine have never been tested in randomized trials in the ACS setting, while a large, retrospective analysis of the CRUSADE registry suggests that morphine administration may be associated with increased mortality in patients with non-ST-segment elevation ACS [8]. Some of the previously listed side effects of morphine could affect the intestinal absorption and thus the pharmacokinetics (PK) and pharmacodynamics (PD) of orally administered drugs which are concomitantly used with morphine. Note that currently all commercially available P2Y12 blockers are oral agents.

In concordance with those facts, it was observed that morphine lags clopidogrel absorption, decreases plasma levels of clopidogrel and its active metabolite, and delays and diminishes antiplatelet effects of this thienopyridine, although this was observed in a group of healthy volunteers [9]. Moreover, Parodi *et al*. in their study comparing the PD of ticagrelor and prasugrel in STEMI patients reported that morphine administration may be associated with impaired antiplatelet effects of both investigated drugs [10].

However, a definitive evidence of a possible interaction between morphine and the novel P2Y12 receptor antagonists may be obtained only in randomized studies. At present, no PK and PD data regarding the concurrent use of morphine and P2Y12 blockers in the ACS setting are available. Therefore, evidence-based verification of morphine’s influence on the PK and PD of ticagrelor and its active metabolite (AR-C124910XX) could provide a valuable insight into the knowledge regarding modern ACS management.

## Methods/design

### Study objectives

The primary objective of the Influence of Morphine on Pharmacokinetics and Pharmacodynamics of Ticagrelor in Patients with Acute Myocardial Infarction (IMPRESSION) study is to test the hypothesis that intravenous administration of morphine prior to ticagrelor administration in patients with AMI alters the plasma concentrations of ticagrelor and its active metabolite.

The secondary objective of this study is to determine whether intravenous administration of morphine prior to ticagrelor administration in AMI patients attenuates the antiplatelet effects of ticagrelor.

Additionally, a predefined subanalysis has been planned to investigate which of the platelet reactivity assessment methods utilized in the study best reflects concentration of ticagrelor and its active metabolite.

### Study design and population

**Figure 1:**
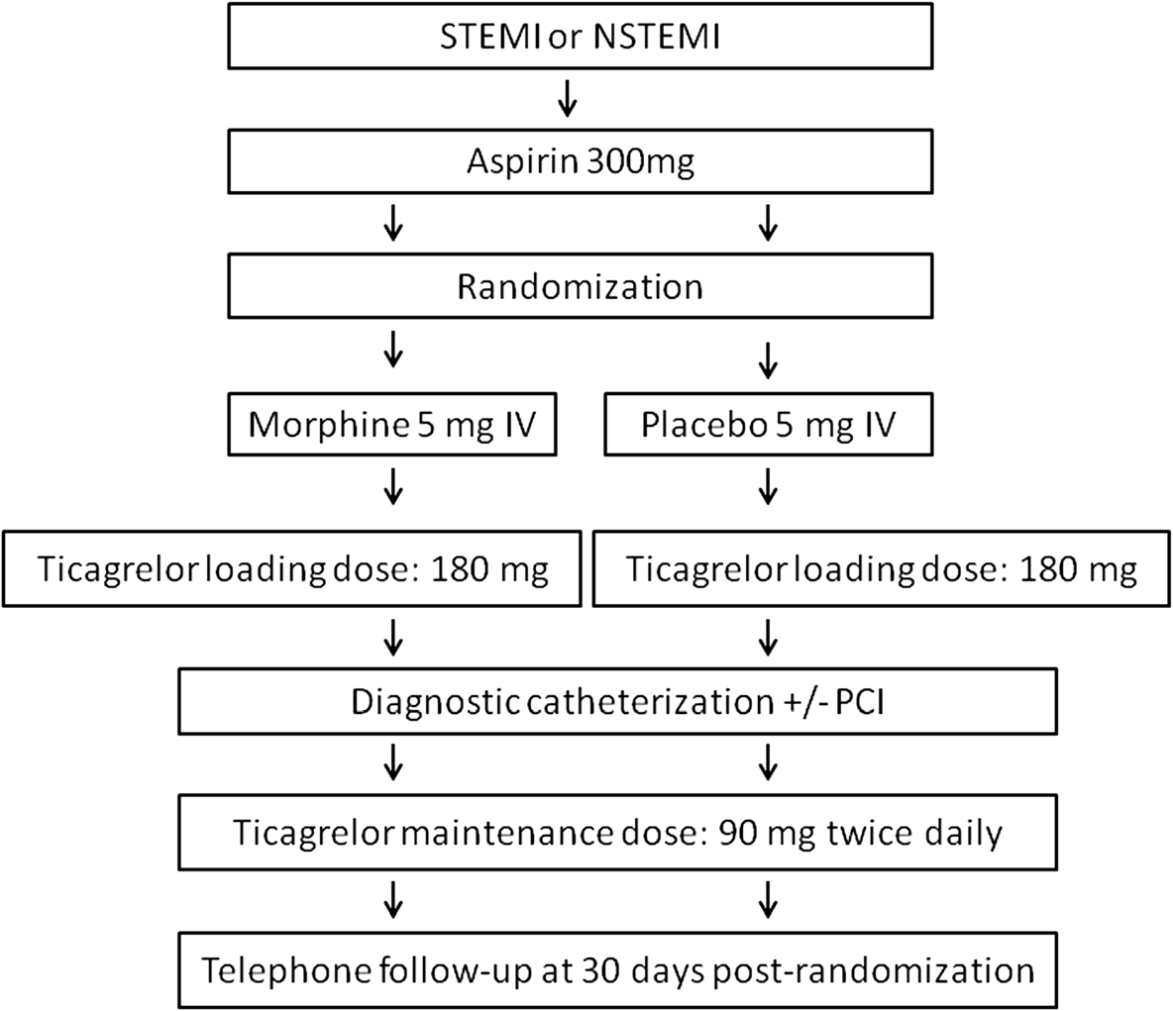
Trial schema for the IMPRESSION study. Legend: IV: intravenous; PCI: percutaneous coronary intervention.

**Figure 2:**
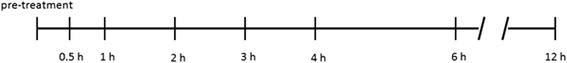
Blood sampling schedule for the IMPRESSION study.

The IMPRESSION study is a phase IV, single center, randomized, double-blind, placebo-controlled, PK and PD clinical trial. The study population includes subjects presenting with AMI, including both STEMI and NSTEMI patients. After admission to the study center (Cardiology Clinic, Dr. A. Jurasz University Hospital, Bydgoszcz, Poland) and confirmation of the initial STEMI or NSTEMI diagnosis, patients will receive orally a 300-mg loading dose of plain aspirin and will be screened for eligibility for the study. The inclusion and exclusion criteria are as listed in Table 1. Eligible patients will be randomly assigned in a 1:1 ratio to one of two study arms. The patients in the intervention arm will receive 5 mg of morphine intravenously (IV) followed by a 180-mg loading dose of ticagrelor, whereas patients in the control arm will obtain 5 mg of a placebo IV followed by a 180-mg loading dose of ticagrelor (Figure 1). Subsequently all patients will undergo a coronary angiography followed by percutaneous coronary intervention (PCI), if necessary. Blood samples for PK and PD assessment will be drawn at eight predefined time points during the first 12 hours after randomization, according to the blood sampling schedule as presented in Figure 2. A telephone follow-up will be conducted at 30 days post-randomization, which will conclude the subject’s participation in the study.

**Table 1:** **Inclusion and exclusion criteria for IMPRESSION study**

Inclusion Criteria	
Provision of informed consent prior to any study-specific procedures	Provision of informed consent for angiography and PCI
Diagnosis of acute STEMI or acute NSTEMI	Male or non-pregnant female, aged 18 to 80 years old
Exclusion Criteria	
Chest pain described by the patient as unbearable or patient’s request for analgesics	History of major surgery or severe trauma (within 3 months)
Prior morphine administration during the current STEMI or NSTEMI	Patients considered by the investigator to be at risk of bradycardic events
Treatment with ticlopidine, clopidogrel, prasugrel, or ticagrelor within 14 days before the study enrollment	Second- or third-degree atrioventricular block during screening for eligibility
Hypersensitivity to ticagrelor	Patient required dialysis
Current treatment with oral anticoagulant or chronic therapy with LMWH	History of asthma or severe chronic obstructive pulmonary disease
Active bleeding	Manifest infection or inflammatory state
History of intracranial hemorrhage	Killip class III or IV during screening for eligibility
Recent gastrointestinal bleeding (within 30 days)	Respiratory failure
History of coagulation disorders	History of severe chronic heart failure (NYHA class III or IV)
Concomitant therapy with strong CYP3A inducers (rifampicin, phenytoin, carbamazepine, dexamethasone, phenobarbital) within 14 days and during study treatment	Concomitant therapy with strong CYP3A inhibitors (ketoconazole, itraconazole, voriconazole, telithromycin, clarithromycin, nefazadone, ritonavir, saquinavir, nelfinavir, indinavir, atazanavir)
Hemoglobin concentration less than 10.0 g/dl	Platelet count less than <100 x10^3/mcl
History of moderate or severe hepatic impairment	Body weight below 50 kg

LMWH: low-molecular-weight heparin; NSTEMI: non-ST-segment elevation myocardial infarction; NYHA: New York Heart Association; PCI: percutaneous coronary intervention; STEMI: ST-segment elevation myocardial infarction.

The study will be conducted in accordance with the principles contained in the Declaration of Helsinki, and the study site received approval from the Local Ethics Committee to conduct the study (Komisja Bioetyczna Uniwersytetu Mikołaja Kopernika w Toruniu przy Collegium Medicum im. Ludwika Rydygiera w Bydgoszczy; study approval reference number KB 111/2014). Each patient will provide a written informed consent to participate in the study.

### Pharmacokinetics

Blood plasma concentrations of ticagrelor and AR-C124910XX will be evaluated using liquid chromatography mass spectrometry in samples obtained in eight predefined time points (Figure 2).

### Pharmacodynamics

**Figure 3:**
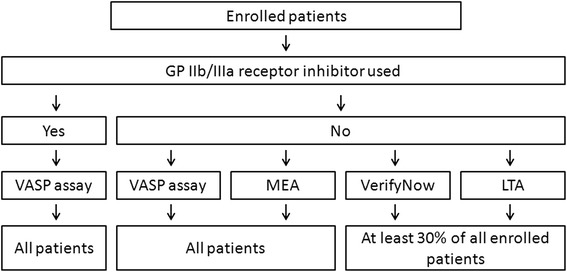
Pharmacodynamic assessment schedule for IMPRESSION study. Legend: GP IIb/IIIa: glycoprotein IIb/IIIa; LTA: light transmission aggregometry; MEA: multiple electrode aggregometry.

Assessment of ticagrelor’s antiplatelet effects will be performed using up to four different methods (Figure 3). A platelet vasodilator-stimulated phosphoprotein (VASP) assay will be applied to all study participants at all predefined time points (Figure 2). Multiple electrode aggregometry (MEA) will be used at all predefined time points for all study participants with the exception of those treated with glycoprotein IIb/IIIa (GP IIb/IIIa) receptor inhibitors. VerifyNow and light transmission aggregometry (LTA) will be applied for at least 30% of all enrolled study participants at all predefined time points. Measurements with VerifyNow and LTA will not be performed in patients treated with GP IIb/IIIa receptor inhibitors. Details regarding methods to be used for the assessment of platelet reactivity were previously described [11-14].

### Treatment protocol and concomitant medications

During their participation in the study, all patients will be treated according to the current ESC guidelines. Administration of GP IIb/IIIa receptor inhibitors will be restricted to bailout situations. Interventional cardiologists will be encouraged to use manual thrombectomy in case of visible thrombus. The choice of the access of the coronary invasive procedure (radial or femoral) and the type of implanted stent (drug-eluting stent [DES] or bare metal stent [BMS]) will be at the discretion of the operator, although high rates of radial access and DES implantation are expected to occur. From the loading dose of ticagrelor until completion of the study, all patients will receive maintenance doses of ticagrelor (90 mg) twice daily. In addition, standard therapy will include aspirin (75 to 100 mg daily), beta blockers, statins, and angiotensin-converting enzyme inhibitors or angiotensin II receptor blockers, if not contraindicated.

### Study endpoints

The primary endpoint of this trial is the area under the plasma concentration-time curve (AUC_(0–12)_) for ticagrelor for the first 12 hours after the loading dose of ticagrelor. Secondary endpoints include AUC_(0–12)_ for AR-C124910XX, maximum concentration (Cmax) of ticagrelor and AR-C124910XX, time to Cmax for ticagrelor and AR-C124910XX, platelet reactivity index (PRI) assessed by VASP assay, area under the aggregation curve assessed by MEA, P2Y12 reaction units (PRU) assessed by VerifyNow, and ADP-induced platelet aggregation assessed by LTA.

### Safety

The following safety endpoints will be recorded: definite stent thrombosis according to the Academic Research Consortium criteria, major and minor, recurrent myocardial infarction according to the Third Universal Definition of Myocardial Infarction, all-cause death, stroke, and transient ischemic attack (TIA) according to definitions used in the PLATO trial, minor and major bleedings according to the BARC, PLATO, and TIMI criteria, dyspnea adverse events according to criteria used in the PLATO trial, bradyarrhythmic events according to criteria used in the PLATO trial.

### Statistics

Since there is no reference study examining the PK of ticagrelor in patients presenting with STEMI or NSTEMI, we decided to perform an internal pilot study of approximately 30 patients (15 patients for each arm) to estimate the final sample size. The means and standard deviations of AUC_(0–12)_ for ticagrelor in the first 33 patients assessed for patients who received morphine and for patients who received the placebo were 6,917 ± 4,920 and 10,379 ± 5,996 ng · h/ml, respectively. Based on these results and assuming a two-sided alpha value of 0.05, we calculated, using the *t*-test for independent variables, that enrollment of 68 patients would provide an 80% power to demonstrate a significant difference in AUC_(0–12)_ for ticagrelor between patients who received morphine prior to a loading dose of ticagrelor and those who received the placebo prior to the ticagrelor loading dose.

Continuous variables will be compared between both study arms by the Student’s *t*-test or the Mann–Whitney *U* test, depending on the presence or absence of the normal distribution (as assessed by the Kolmogorov-Smirnov test). Proportions will be compared by the Fisher exact test or chi-square test when appropriate. Pharmacokinetic calculations will be performed using a dedicated software.

## Discussion

The IMPRESSION study is a phase IV, single center, randomized, double-blind, placebo-controlled clinical trial that assesses the influence of morphine on the PK and antiplatelet effects of ticagrelor in patients with myocardial infarction. This trial will provide important information regarding the impact of morphine, the analgesic which is most commonly used in patients with AMI, on the absorption of ticagrelor in subjects presenting with STEMI and NSTEMI. Furthermore, the study could specify the suspected connection between alterations in ticagrelor absorption and modification of its antiplatelet activity. A disclosure of such drug-drug interaction in AMI patients could supply a significant evidence-based data source regarding concurrent use of ticagrelor and morphine in STEMI and NSTEMI treatment.

## Trial status

The first patient was enrolled in August 2014. Initially the IMPRESSION study was planned to include STEMI patients only. However, on 9 September 2014, after the approval of the Local Ethics Committee, the trial population was expanded to include subjects with NSTEMI as well. By 8 January 2015, 33% of planned patients were successfully included in the study. The baseline characteristics of the enrolled patients are presented in Table 2.

**Table 2:** **Baseline characteristics**

	**STEMI (n = 22)**	**NSTEMI (n = 11)**	**Total (n = 33)**
Age in years, median	62.6	62.4	62.5
Age ≥ 70	9 (40.9%)	2 (18.2%)	9 (27.3%)
Female	5 (22.7%)	2 (18.2%)	7 (21.2%)
Hypertension	7 (31.8%)	8 (72.7%)	15 (45.5%)
Diabetes mellitus	5 (22.7%)	2 (18.2%)	7 (21.2%)
Dyslipidemia	19 (86.4%)	8 (72.7%)	27 (81.8%)
Current smoker	9 (40.9%)	6 (54.5%)	15 (45.5%)
Prior MI	1 (4.5%)	3 (27.3%)	4 (12.2%)
Prior PCI	1 (4.5%)	5 (45.5%)	6 (18.2%)
Prior CABG	0	0	0
BMI in kg/m^2^	28.4	27.4	28.1

BMI: body mass index; CABG: coronary artery bypass surgery; MI: myocardial infarction.

## Abbreviations

ACCF/AHA: American College of Cardiology Foundation and American Heart Association
ACS: acute coronary syndromes
AMI: acute myocardial infarction
AUC: area under the curve
BMS: bare metal stent
Cmax: maximum concentration
DES: drug-eluting stent
ESC: European Society of Cardiology
GP IIb/IIIa: glycoprotein IIb/IIIa
IV: intravenously
LTA: light transmission aggregometry
MEA: multiple electrode aggregometry
NSTEMI: non-ST-segment elevation myocardial infarction
PCI: percutaneous coronary intervention
PD: pharmacodynamics
PK: pharmacokinetics
PRI: platelet reactivity index
PRU: P2Y12 reaction units
STEMI: ST-segment elevation myocardial infarction.
